# P-1238. Assessment of Body Weight upon PK of Olorofim in Patients with Invasive Mould Infections

**DOI:** 10.1093/ofid/ofae631.1420

**Published:** 2025-01-29

**Authors:** Karen Cornelissen, John H Rex, Daniela Zinzi, Hal Galbraith, Howard Burt

**Affiliations:** F2G, Manchester, England, United Kingdom; F2G, Limited, WELLESLEY HILLS, MA; F2G, Manchester, England, United Kingdom; IQVIA Inc, Overland Park, Kansas; Certara UK Ltd (Simcyp Division), Sheffield, England, United Kingdom

## Abstract

**Background:**

Olorofim (OLO) is a novel antifungal active vs. *Aspergillus* (including azole-resistant strains), resistant moulds (e.g., *Lomentospora prolificans [LoPro]*), and dimorphic moulds. Potential impact of bodyweight upon systemic exposure was explored using pharmacokinetic (PK) data from a Phase IIb study and PB (physiologically-based) PK modelling.

**Figure:**
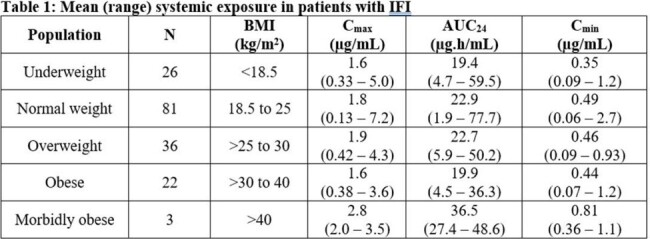

**Methods:**

Adult patients in the Phase IIb study (N=203) received OLO (30 mg tablets) at a nominal dose of 90 mg BID and OLO PK profiles were determined over 1 or 2 dosing intervals after 7 to 14 days dosing, with an additional 8 to 9 scheduled pre-dose samples taken over the initial 84 days.

A PBPK model was developed using both *in vitro* and *in vivo* data where oxidative cytochrome P450 (CYP)-mediated metabolism was determined to be the major pathway of olorofim clearance. The model was verified via demonstration of good independent recovery of observed systemic exposure in the presence and absence of CYP inhibitors and inducers and applied to the prediction of exposure in obese and morbidly obese subjects.

**Results:**

Of the 168 patients with Invasive Fungal Infection (IFI), known body mass index (BMI) and full PK data over a dosing interval, 48% were normal body weight, 13 to 21% were classed as underweight, overweight or obese, with 1.8% classified as morbidly obese. Steady state systemic exposure (as determined by non-compartmental PK analysis) was similar across all BMI ranges (Table 1), apart from morbidly obese. The apparently higher mean exposure for morbidly obese is based on N=3 and values fall within the range seen for all other groups.

These results agree with PBPK modelling which predicted negligible to mild increases in total plasma olorofim AUC and C_max_ values in obese subjects (geometric mean ratios for normal to obese of up to 1.25-fold), whereas a negligible change was predicted in morbidly obese subjects (which further suggests that the observed elevation in exposure in morbidly obese is artefactual likely driven by limited number for this subject population).

**Conclusion:**

As systemic exposure in adults with IFI was similar across a range of BMIs, dose adjustment based on body weight is not required when treating obese or morbidly obese patients.

**Disclosures:**

**Karen Cornelissen, PhD**, F2G: Stocks/Bonds (Private Company) **John H. Rex, MD**, F2G: Employee **Daniela Zinzi, MD. Infectious Diseases Specialist**, VP Clinical R&D at F2G: Stocks/Bonds (Private Company)

